# Is a preoperative multidisciplinary team meeting (cost)effective to improve outcome for high-risk adult patients undergoing noncardiac surgery: the PREPARATION study—a multicenter stepped-wedge cluster randomized trial

**DOI:** 10.1186/s13063-023-07685-3

**Published:** 2023-10-11

**Authors:** Jacqueline E. M. Vernooij, Romijn M. Boerlage, Carine J. M. Doggen, Benedikt Preckel, Carmen D. Dirksen, Barbara L. van Leeuwen, Rutger J. Spruit, Suzanne Festen, Hanneke van der Wal-Huisman, Jean P. van Basten, Cor J. Kalkman, Nick J. Koning, PREPARATION study investigators, Koene van der Sloot, Esther M. Dias, Jasper E. Kal, Marjolein C. O. van den Nieuwenhuyzen, Manuela di Biase, Martin Hagenaars, Bies Oedairadjsingh, Taco van den Ende, Michel Timmerman, Zjuul Segers, Dominique H. P. A. M. Schoester, Kristy M. J. Vons, A. Filius, Wim van Harten, Rudolf W. Poolman, Michel M. P. J. Reijnen, Peter G. Noordzij, Barbara C. van Munster

**Affiliations:** 1 Department of Anesthesiology, Rijnstate Hospital, Arnhem, The Netherlands; 2 Department of Anesthesiology, University Medical Centre Utrecht, Utrecht, The Netherlands; 3 Department of Health Technology and Services Research, Technical Medical Centre, University of Twente, Enschedé, The Netherlands; 4 Clinical Research Center, Rijnstate Hospital, Arnhem, The Netherlands; 5 Department of Anesthesiology, Amsterdam UMC Location AMC, Amsterdam Public Health, Quality of Care, Amsterdam Cardiovascular Science, Diabetes & Metabolism, Amsterdam, The Netherlands; 6 Care and Public Health Research Institute, Maastricht University Medical Center, Maastricht, The Netherlands; 7 Department of Surgery, University of Groningen, University Medical Center Groningen, Groningen, The Netherlands; 8 Department of Anesthesiology, University of Groningen, University Medical Center Groningen, Groningen, The Netherlands; 9 University Center for Geriatric Medicine, University Medical Center Groningen, Groningen, The Netherlands; 10 Department of Urology, Canisius-Wilhelmina Hospital, Nijmegen, The Netherlands

**Keywords:** Preoperative multidisciplinary team, High-risk, Complications, Costs, Quality of life, Risk tools, Frailty, Mortality, Patient’s preferences, Serious adverse events, Stepped-wedge randomized cluster design, Cost-effectiveness, Health technology assessment

## Abstract

**Background:**

As a result of increased life expectancy and improved care for patients suffering from chronic disease, the number of patients with multimorbidity requiring surgical intervention is increasing. For complex surgical patients, it is essential to balance the potential benefits of surgical treatment against the risk of permanent loss of functional capacity and quality of life due to complications. European and US guidelines on perioperative care recommend preoperative multidisciplinary team (MDT) discussions for high-risk noncardiac surgical patients. However, the evidence underlying benefits from preoperative MDT meetings with all relevant perioperative specialties present is limited. The current study aims to investigate the effect of implementation of preoperative MDT discussions for high-risk patients undergoing noncardiac surgery on serious adverse events.

**Methods/design:**

PREPARATION is a stepped-wedge cluster randomized trial in 14 Dutch hospitals without currently established preoperative MDT meeting. The intervention, preoperative MDT meetings, will be implemented sequentially with seven blocks of 2 hospitals switching from control (preoperative screening as usual) to the intervention every 3 months. Each hospital will be randomized to one of seven blocks. We aim to include 1200 patients. The primary outcome is the incidence of serious adverse events at 6 months. Secondary outcomes include (cost)effectiveness, functional outcome, and quality of life for up to 12 months.

**Discussion:**

PREPARATION is the first study to assess the effectiveness of a preoperative MDT meeting for high-risk noncardiac surgical patients in the presence of an anesthesiologist. If the results suggest that preoperative MDT discussions for high-risk patients are (cost)-effective, the current study facilitates implementation of preoperative MDT meetings in clinical practice.

**Trial registration:**

ClinicalTrials.gov NCT05703230. Registered on 11/09/2022.

**Supplementary Information:**

The online version contains supplementary material available at 10.1186/s13063-023-07685-3.

## Introduction

As a result of increased life expectancy and improved care for patients with chronic diseases, the number of frail patients and patients with multimorbidity requiring surgical interventions is rising [1, 2]. Frail patients and patients with multimorbidity undergoing noncardiac surgery are at high risk for postoperative complications and hospital readmissions [3–7]. Surgical treatment is often performed with the dual goals of improving quality of life as well as survival. However, there is a crucial balance between the potential benefits of surgery and the risk of permanent loss of functional capacity and diminished quality of life from surgical complications. Patients may prioritize quality of life over receiving treatment to extend their survival, even if the treatment offered follows current guidelines [8]. For the individual medical specialist faced with these high-risk patients, it may be challenging to determine whether a technically feasible procedure is also in the patient’s best interest.

Multidisciplinary team meetings accommodate discussions between various specialists regarding optimal and individualized treatment plans. In several medical specialties, MDT meetings are recommended and already frequently established [9, 10]. Oncological MDT meetings, also known as “tumor boards,” have been widely studied, describing potential benefits on patient outcomes [11]. Many high-risk patients scheduled for noncardiac surgery, however, have not been discussed in a multidisciplinary team meeting. Additionally, tumor boards usually focus on technical aspects of the disease and the surgical procedure and may be hindered by an excessive caseload, time pressure, and lack of patient-specific information [12, 13]. Furthermore, the anesthesiologist with specific knowledge on perioperative risks and care, is often absent. A discussion of patient-specific benefits and risks of the planned surgery may therefore be lacking, limiting the potential positive effect of discipline-specific MDT meetings on patient outcome [14].

European and US guidelines on perioperative care recommend preoperative multidisciplinary discussion for high-risk noncardiac surgical patients [15–17]. However, the added value of preoperative MDT discussions is currently based on the consensus of expert opinion, registries, and small or retrospective studies [5, 18, 19]. A previous study where high-risk patients planned for noncardiac surgery were selected for preoperative MDT discussions by the anesthesiologist at the preoperative clinic, showed that 43% of high-risk patients did not undergo surgery after discussion in a preoperative MDT meeting [5]. Cancelation of surgery was related to either the multidisciplinary team consensus-based advice or the patient’s own decision after reconsidering the surgical harm-benefit ratio.

The primary aim of this stepped-wedge cluster randomized trial (SW-CRT) is to assess whether preoperative MDT discussions for high-risk patients undergoing noncardiac surgery lead to a decrease in serious adverse events (SAEs) after 6 months. Secondly, the current trial will assess the cost-effectiveness of implementing a preoperative MDT meeting for high-risk noncardiac surgical patients compared to current preoperative screening practices. Furthermore, we aim to assess the effect of preoperative MDT discussions on survival, quality of life, and functional outcome, as well as the patient’s decision regret [20]. In addition, the quality of decision-making in these MDT meetings and facilitators and barriers for implementing MDT meetings in clinical practice will be investigated.

## Methods

### Study setting

This multi-center stepped-wedge cluster randomized trial (NCT05703230—ClinicalTrials.gov), the PREPARATION study, will be conducted in 14 academic, teaching, and general hospitals in The Netherlands (Additional file 2). High-risk noncardiac surgical patients are identified at the preoperative anesthesia clinic in each hospital. The intervention (MDT meeting including anesthesiologic expertise) will be implemented sequentially to 14 hospitals (clusters) without such an MDT meeting already established. This trial protocol uses the Standard Protocol Items: Recommendations for Interventional Trials (SPIRIT) reporting guidance [21]. The WHO dataset on this study is available as Additional file 3.

### Study population

Anesthesiologists screen all patients scheduled for noncardiac surgery in the participating hospitals to identify those eligible for participation in this trial. Patients are eligible if they meet the following inclusion criteria:

-  ≥ 18 years of age
- American Society of Anesthesiology (ASA) physical status score ≥ 3
- Clinical Frailty Scale ≥ 4 [22]
- Planned for elective or semi-elective non-cardiac surgery
- As stated by the 2010 Dutch preoperative guideline [23]:
  - ∘ Doubt by the surgeon, anesthesiologist or patient regarding the harm-benefit ratio of the surgical procedure
  - ∘ Doubt if the correct measures were taken to limit the perioperative risk as much as possible
  - ∘ Doubt if the patient agrees with the surgical and anesthetic plan and the expected risks

The exclusion criteria are:

- No informed consent
- Emergency surgery
- The impossibility to communicate with the patient directly or through a third party, such as a relative or an interpreter
- Proposed surgical procedure for which a preoperative MDT meeting, similar to the current study intervention, already exists in that hospital at the start of the study

### Intervention

The intervention consists of implementation of preoperative MDT discussions for high-risk noncardiac surgical patients. In the preoperative MDT meeting, a patient is discussed among at least a surgeon, an anesthesiologist, and one or more medical consultant(s) and/or specialized nurses. Relevant medical consultants can be invited specifically based on the patient’s comorbidities. During the meeting, the attendees will review the technical aspects of the scheduled surgery, estimate the harm-benefit ratio of the procedure, evaluate the impact of existing comorbidities, and discuss the patient’s expectations and preferences.

At least the following questions should be discussed:

- Is the proposed surgical intervention the most appropriate care for this patient and what are the alternative treatments?
- Is the harm-benefit ratio of the proposed surgical intervention acceptable for this patient?
- Should the patient’s condition be optimized before undergoing the proposed surgical intervention?

To facilitate the implementation of preoperative MDT meetings, a toolbox is provided to the participating hospitals to guide discussion with regard to health condition, patient preferences in life, and treatment options (Additional file 4). This toolbox aids in the development of a comprehensive treatment plan. In addition, the Outcome Prioritization Tool (OPT) will be used preoperatively to systematically collect information regarding patients’ goals and preferences in life [24].

### Outcome

The primary outcome is the incidence of serious adverse events (SAEs) [25] at 6 months, defined as Grade 3 or more on the Clavien-Dindo classification following surgical intervention [26]. In the case of non-surgical management, serious adverse events will be graded accordingly, such as events necessitating endoscopic or surgical intervention, single or multiple organ failure, or death [26].

Secondary outcomes (Table 1) include:

- Cost-effectiveness from a societal and healthcare perspective, resource use measured by a patient cost questionnaire and via hospital electronic health records, health-related quality of life measured with the EQ-5D-5L for the construction of quality-adjusted life years (QALYs) [27] 
- Functional status of the patient, measured by the 12-item WHO Disability Assessment Schedule 2.0 (WHODAS 2.0) [28]
- Patients’ experienced quality of life assessed by the abbreviated World Health Organization Quality of Life (WHOQOL BREF) [29]
- Patients’ regret about the treatment decision measured by the Decision Regret Scale [20]
- Survival
- The attendance and self-assessed performance of MDT meetings are evaluated using a form that assesses various aspects such as structure, duration, cases reviewed, attending healthcare staff, medical specialty, initial queries, and treatment choices.
- Quality of MDT meetings by observations of multidisciplinary team discussion and decision-making by an independent, non-participant observer, using the MDT-MODe of decision-making (MDT-MODe) in a subset of MDT meetings [30, 31]
- Delay between preoperative screening, preoperative MDT meeting, and the surgical procedure
- Facilitators and barriers to organize preoperative MDT meetings

**Table 1:** Primary and secondary outcomes, data sources, and used instrument/source of information

Outcome	Type of data source	Instrument/source
Serious adverse events	Registration	EHR
Functional status	Questionnaire	WHODAS 2.0 [28]
Health-related quality of life	Questionnaire	EQ-5D-5L} [27]
Quality of life	Questionnaire	WHOQOL BREF [29]
Decision regret	Questionnaire	Decision Regret Scale [20]
MDT characteristics	Registration	CRF
Perioperative management	Registration	CRF
MDT decision-making and facilitators and barriers	Observations and interviews	MDT-MODe [30]
Survival	Registration	EHR
Resource use	Questionnaire and registration	iMCQ and iPCQ: CRF: EHR [32]
Delay between preoperative screening, preoperative MDT, and surgery	Registration	CRF

*EHR* electronic health record, *WHODAS 2.0* World Health Organization Disability Assessment Schedule 2.0, *EQ-5D-5L* quality of life in 5 dimensions, *WHOQOL BREF* World Health Organization Quality of Life, short version, OPT Outcome Prioritization Tool, *CRF* case report form, *MDT-MODe* multidisciplinary team mode of decision-making, *iMCQ* iMTA (institute for Medical Technology Assessment) Medical Consumption Questionnaire, *iPCQ* iMTA Productivity Cost Questionnaire

### Design

The PREPARATION study is designed as a stepped-wedge cluster randomized trial and will be executed in 14 hospitals without a currently established preoperative MDT meeting for high-risk noncardiac surgical patients [33]. The current trial employed a stepped-wedge design to address ethical concerns. This decision was based on the appreciation for existing MDT meetings among patients and physicians and the moral dilemma posed by denying patients the opportunity to have their cases discussed in preoperative MDT meetings in hospitals where such meetings are already standard practice [4, 5, 34–36]. Therefore, participating hospitals were selected based on the absence of established preoperative MDT meetings. At the start of the trial, each participating hospital performs preoperative screening and management as usual without preoperative MDT discussions (phase 1). At the crossover time points, two study centers switch from the control condition to the implementation of preoperative MDT discussions (phase 2). After 24 months, all hospitals have implemented a preoperative MDT meeting (i.e., all hospitals will be exposed to the intervention).

**Fig. 1:**
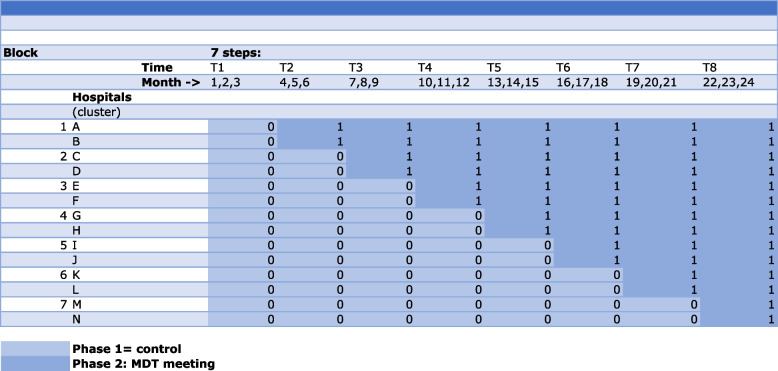
Diagram of the stepped-wedge cluster randomized trial design. Participating hospitals are randomized to represented rows A to N and are divided in 7 blocks of 2 hospitals. T1–T8: time periods of 3 months. All patients will have a follow-up time of 12 months

**Fig. 2:**
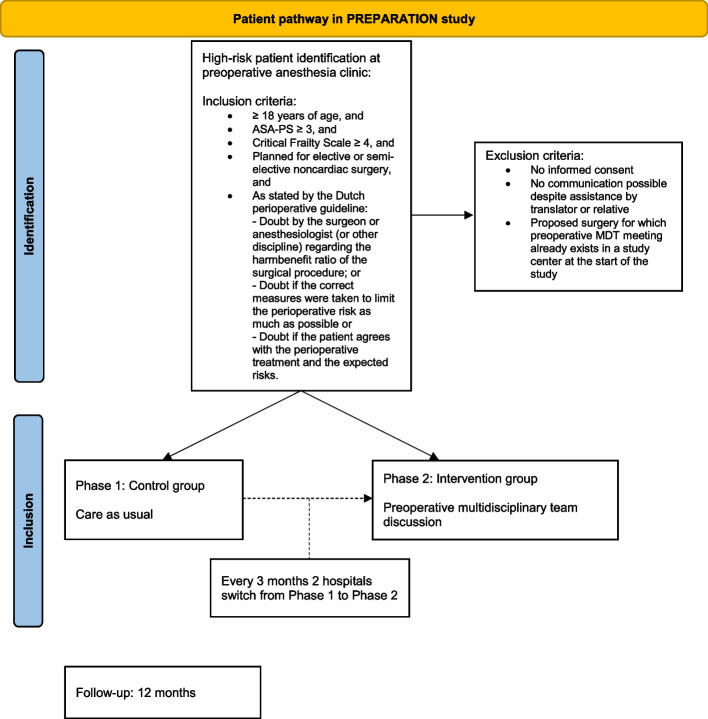
Individual patient pathway during participation in the PREPARATION study. ASA-PS, American Society of Anesthesiology Physical Status; MDT, multidisciplinary team [37]

The design pattern matrix (Fig. 1) illustrates the trial design. In total, there will be 7 blocks, each consisting of two hospitals. Each hospital will be randomized to one of the seven sequences prior to the start of the study by an independent person from the coordinating center (Fig. 1). The individual patient flow, either as a patient in phase 1 (control) or as a patient in phase 2 (intervention), is shown in Fig. 2. Computer-generated lists of random numbers will be used to randomly assign the hospitals to one of the sequences (2 hospitals per sequence/block) for the 7 fixed number of points in time of crossover (steps). Furthermore, we will stratify on the size of the hospital (small versus large). This ensures that the control and intervention are balanced on the type and size of the hospital. Each hospital will be informed of its own crossover date 3 months prior to this time point to ensure ample time for preparing the implementation of the MDT. All patients will be individually followed in time until 1 year from the day of surgery or the day of the preoperative MDT meeting in case of nonsurgical management.

### Recruitment

Consecutive high-risk noncardiac surgical patients will be assessed for potential eligibility at the preoperative assessment by the anesthesiologist and asked to participate if they fulfill the inclusion criteria. Eligible patients will receive written and video information about the study. Written informed consent will be obtained by a trained (research) nurse or a physician before surgery (control; phase 1) or before the MDT meeting (intervention; phase 2). To facilitate the inclusion of patients with limited health literacy in our study, we incorporated measures to guarantee that information about the study, questionnaires, and informed consent were written at a suitable language level (level B1).

Each hospital is estimated to include ten patients on average per 3 months. As hospitals vary in size and do not serve the same number of surgical patients, each hospital includes a different number of patients during each period of 3 months. Patients can withdraw consent at any time for any reason if they wish to do so. Data from patients who withdraw will be used in the analysis until the date of withdrawal, unless the patient states otherwise.

### Statistical analysis plan

Primary outcome: Characteristics of the hospitals and patients will be summarized by group of randomization. Continuous variables will be described using means with standard deviations (SD) or medians with quartile ranges. We will check for imbalances between the control and intervention groups. Differences in continuous variables will be assessed using t-tests or non-parametric tests (Wilcoxon signed rank test) whichever is appropriate. Categorical variables will be described using numbers and percentages. Differences will be assessed using chi-square tests or Fisher’s exact tests. A *p*-value lower than 0.05 will be considered a statistically significant difference. We will determine the crude rate of SAEs for the control and intervention period. Data will be analyzed according to the intention to treat principle. Additionally, a per-protocol analysis will be performed based on whether the patients’ case was discussed in a preoperative MDT meeting or not. Differences in SAEs will be analyzed using a generalized linear mixed model (GLMM), with a random effect for hospital (accounting for the clustering), adjusted for the size of the hospital (used in the constrained randomization), and for the possible confounding effect of the calendar period. The result will be expressed as the risk difference and odds ratio with the appropriate 95% confidence intervals. In case there will be differences in prognostic variables in the control and intervention group, these potential confounding variables will be added to the GLMM model. Results for unadjusted (except for clustering effect, calendar time, and size of hospital) and adjusted (by potential confounding variables) will be reported. Secondary outcomes: Analyses of the secondary outcomes for categorical variables will also be performed using GLMM, in a similar model as the one used in the analysis of the primary outcome, with adjustment for other variables if indicated. In case of counts, such as the number of SAEs, GLMM will be used assuming a Poisson-dependent variable. These results will be reported as adjusted relative risk to summarize the difference between the control and intervention periods. For numeric outcome variables linear mixed models with adjustment for other variables will be used. The MDT meeting’s execution may be refined over time. Therefore, time-by-treatment effect interactions will also be analyzed. Subgroup analyses: The study will execute exploratory subgroup analyses based on factors such as age (< 70, ≥ 70 years), size of the hospital (small vs large), and intent of surgery (life-extending, functional, palliative, relief of pain or other complaints, other). Data analysis: Data analysis will be performed using the software package IBM SPSS Statistics, version 28 for Citrix (SPSS, Inc., Chicago, IL, USA) and R statistical language (https://www.r-project.org). All structured interviews will be audio recorded and transcribed verbatim. The transcripts will be analyzed by two independent coders, using thematic inductive analysis. Coders will meet on several occasions to compare their findings and differences will be discussed until consensus is reached.

### Sample size

Statistical analysis determined that a sample size of 14 clusters, or around 1120 patients, would be needed to identify a 15.5% decrease in clinically significant SAEs (Clavien-Dindo ≥ 3). The results showed that this sample size would achieve 87% power with a two-sided test at a 5% significance level. These calculations were based on pilot data that showed a 43% adverse event rate in the control group and a 27.5% rate in the intervention group [5]. We used the Shiny CRT calculator: https://clusterrcts.shinyapps.io/rshinyapp to calculate the sample [38]. No literature is available to guide potential values of the period intra-cluster coefficient (ICC). We assumed that the outcomes of different patients from the same hospital would not change over different periods, apart from the difference in the control condition period and the MDT meeting period. However, we chose a non-exchangeable correlation structure (with a two-period decay) in our sample size calculation to consider an unexpected decline. For the ICC, we used a moderate value of 0.1, and for the cluster auto-correlation (CACs), a value of 0.8. Lower values of the ICC lead to a higher power. Furthermore, we used the T-distribution as we have relatively small samples. We expect an average of 20 new high-risk noncardiac patients in each block. We assume that no correlation exists between hospitals. As we expect patients to drop out we will include 1200 patients in this study (± 7% dropout = 1120).

### Economic evaluation

A trial-based economic evaluation will be performed from both a societal—and healthcare perspective, with a time horizon of 12 months. First, a cost-utility analysis from a societal perspective will calculate the incremental societal costs per quality-adjusted life years (QALY). For this purpose, the EQ-5D-5L will be converted to utilities and hence to QALY’s. Second, a cost-effectiveness analysis from a healthcare perspective will be performed, in which incremental healthcare costs per SAE prevented will be calculated. Data will be analyzed according to “intention to treat.” Standard sensitivity- and bootstrap analyses will be performed to quantify the uncertainty of the costs and cost-effectiveness outcomes. The results of this analysis will be presented in cost-effectiveness planes and cost-effectiveness acceptability curves, showing for a range of threshold values for cost-effectiveness the probability that preoperative MDT is cost-effective. The cost calculation will be performed according to the Dutch guidelines for cost research [39]. Societal costs, consisting of healthcare costs, and costs outside the healthcare sector such as productivity costs, patient and family costs, will be based on actual resources used. Resource use will be measured in natural units and will be valued in monetary terms by multiplying these units by the cost-price per unit. If available, standardized, national cost prices will be used [39]. All hospital resource use (e.g., type of anesthesia, type of surgery, hospital/intensive care days, outpatient visits, re-admissions, and medical procedures) including preoperative MDT discussion or standard preoperative screening, up to 12 months will be recorded on a patient level by means of case report forms (based on hospitals’ electronic patient records). As no detailed cost-price is available regarding the care as usual and regarding the preoperative MDT meeting, a small time-and-motion study (registering duration of the MDT meeting and MDT attendants) will be performed in a selection of patients to obtain an average price-estimate of this activity. Healthcare and non-healthcare costs outside the hospital will be collected by means of a standardized cost questionnaire with a recall period of 3 months. Absence of work, i.e., productivity costs will be calculated by using the friction cost method, as recommended by the Dutch guidelines for cost research [39].  Parts of the MCQ and iPCQ will be incorporated into the cost questionnaire [32, 40]. Where applicable, cost and effectiveness calculations will take into account clustering and time effects related to the stepped-wedge cluster randomized trial design [41]. Discounting of costs and effects is not applicable due to the time horizon of 12 months.

### Data collection and management

**Fig. 3:**
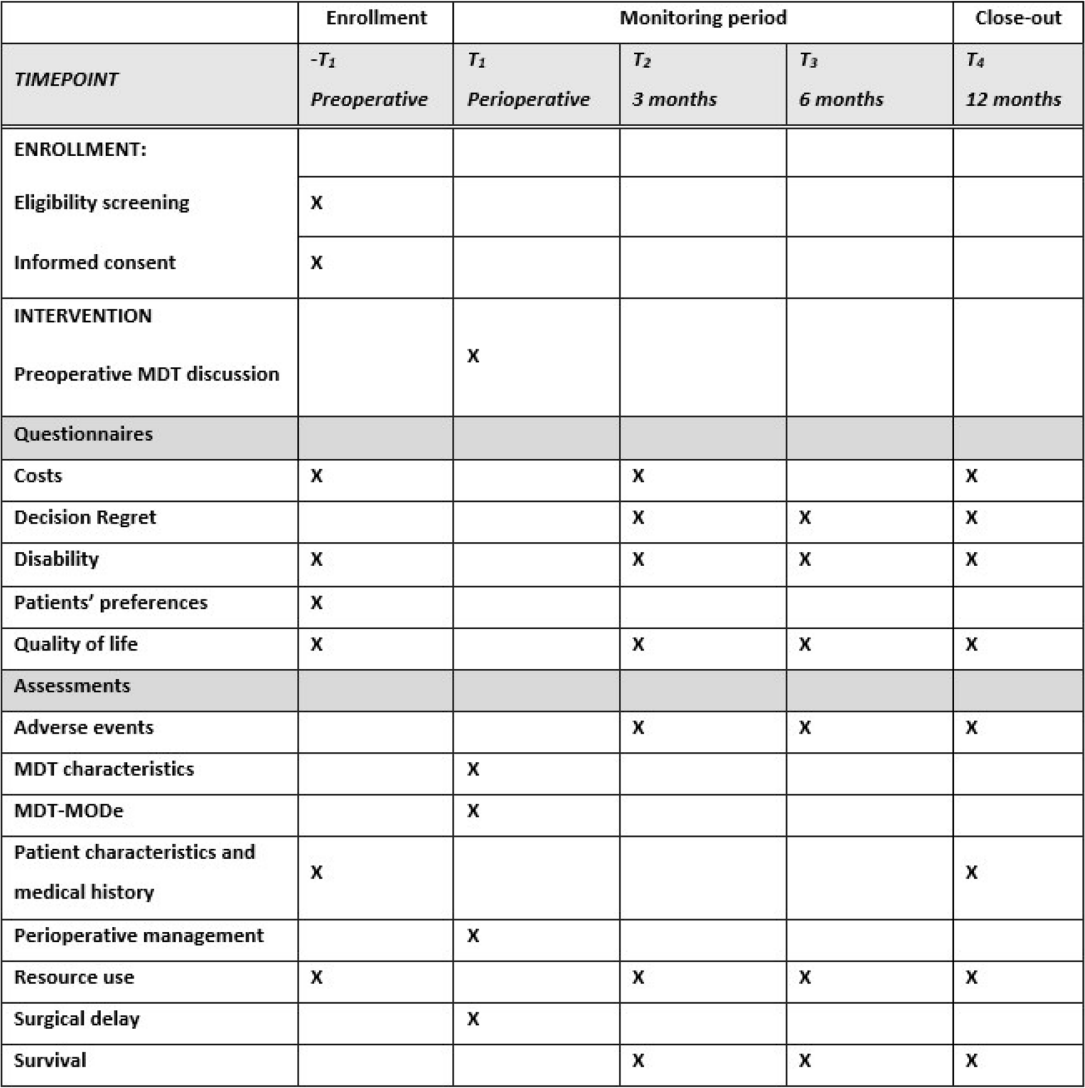
PREPARATION study schedule of enrollment, questionnaires, and assessments. * − t_*1*_, preoperative phase; *t*_*1*_, perioperatively; *t*_*2*_, 3-month follow-up; *t*_*3*_, 6-month follow-up; *t*_*4*_, 12-month follow-up; *MDT*, multidisciplinary team; *MDT-MODe*, MDT-mode of decision-making

Data on primary and secondary outcomes will be collected from patient questionnaires, the case report form, electronic health records, observations of MDT meetings, discussions with patients regarding their preferences, and from interviews with patients and healthcare professionals (Table 1 and Fig. 3). Apart from the MDT meeting observations this process is identical for both study phases, irrespective of control or intervention group. Data of individual participating patients will be provided with a subject identification code. The code will be numbered in order of patient entrance in the study. All collected data are protected according to the data protection standards of The Netherlands and the European Union. All data is entered into an online database: Research Manager (6.8, Research Manager, Deventer, The Netherlands). Only local hospital investigators, the project leader, research coordinator, and two PhD students have access to patient data codes, safeguarded by the principal investigator. EHR and general practitioner data will be collected into a separate database with subject codes and stored on a secure network drive. Questionnaire data will also be collected in the same database. Data will be kept for up to 15 years and only authorized organizations have access. Follow-up questionnaires will be collected digitally by web-based questionnaires software (Research Manager), provided in a booklet by mail, or collected through (telephone) interviews by a trained researcher. Missing data will be evaluated and imputation will be used if necessary.

### Monitoring and dissemination

The PREPARATION study will be conducted in compliance with relevant Dutch laws and regulations governing the conduct of research involving human subjects, such as the Medical Research Involving Human Subjects Act and Medical Treatment Contracts Act. Formal auditing is not required. An external monitor will assess all study sites (hospitals) once, with subsequent follow-up assessments as needed.

The findings of the current cost-effectiveness study will inform the adoption or abandonment of current preoperative MDT meeting practices through guideline adaptation on a national level. The recommendations and guidelines may be modified based on the results of this study. Project members will disseminate the study results through publications and standard channels such as presentations at national and international congresses, symposia, and other scientific meetings.

## Discussion

If the anesthesiologist, another healthcare professional, or the patient harbors serious doubt regarding the potential advantages and risks of the suggested surgery procedure for high-risk patients, the Dutch perioperative guideline recommends scheduling a preoperative MDT meeting [23]. The study’s inclusion criteria were established based on the Dutch guideline, as checklists or risk scores could not fully replace the estimation of harm-benefit ratios. Identification of patients at high risk of an adverse outcome after surgery with risk models is difficult and underscores the critical role of healthcare professionals in identifying patients who may benefit from preoperative MDT discussions [42, 43]. Previous research has demonstrated that preoperative MDT discussions with anesthesiologist’s subjective selection of patients significantly impact patient care, underscoring the anesthesiologist’s vital role perioperatively and the significance of multidisciplinary healthcare during the perioperative phase for high-risk noncardiac surgical patients [5, 19].

The current trial boasts a significant advantage in that, should the preoperative multidisciplinary team (MDT) meetings prove successful in mitigating serious adverse events (SAEs), enhancing quality of life, and optimizing cost-effectiveness, the study’s design will facilitate implementation across the fourteen participating hospitals. Additionally, extensive experience with implementation of preoperative MDT meetings will have been gained at the end of the study. This experience may be used to facilitate implementation of preoperative MDT discussions for high-risk noncardiac surgical patients in other hospitals which do not have regular preoperative MDT meetings installed yet.

Preoperative MDT meetings, the intervention of the current study, may show a large variability between hospitals in both the course of the discussion and the decisions made. In the current study, hospitals are assisted with preparing the implementation of the MDT meetings and provided with feedback after observation of early MDT meetings, in order to maximize quality and uniformity of MDT discussions between different hospitals. Moreover, valuable information about organization, attendance, quality of discussion and decision-making during these preoperative MDT meetings will be acquired during the study. Furthermore, it needs to be clarified how the patients’ health situation and preferences can be optimally integrated into the discussion during a preoperative MDT meeting [44].

Given the profound increase in multimorbidity in the current population, it has become evident that single disease-oriented management programs are less effective in providing high-quality care compared to patient-centered approaches [1]. Preoperative multidisciplinary team discussions for high-risk surgical patients may add patient-centered quality care to complex preoperative decision-making and perioperative care.

### Trial status

Protocol version: 2.5; October 18, 2022.

Trial start date: November 1st, 2022; Trial completion: November 1st, 2025.

### Supplementary Information

**Additional file 1. **The PREPARATION study investigators.

**Additional file 2. **Participating hospitals, all in The Netherlands.

**Additional file 3. **WHO trial dataset.

**Additional file 4. **Tool box for implementation of multidisciplinary team meeting.

**Additional file 5. **WHO trial registry data set.

**Additional file 6. **

**Additional file 7. **

**Additional file 8. **

## Data Availability

The full protocol, data sets, and statistical code utilized in the present study will be made accessible on request after agreement has been received from the project group through the corresponding author.

## Abbreviations

GLMM: Generalized linear mixed model
EHR: Electronic health record
ICC: Intra-cluster coefficient
MDT: Multidisciplinary team
MDT-MODe: MDT-Meeting Observational Tool
OPT: Outcome Prioritization Tool
QALY: Quality adjusted life year
SAE: Serious adverse event
SW-CRT: Stepped wedge cluster randomized tool
WHODAS 2.0: World Health Organization Disability Assessment Schedule 2.0
WHOQOL – BREF: World Health Organization Quality of Life Questionnaire – short version
