# Supplementary material for: Is a preoperative multidisciplinary team meeting (cost)effective to improve outcome for high-risk adult patients undergoing noncardiac surgery: the PREPARATION study—a multicenter stepped-wedge cluster randomized trial
Source: Trials. 2023 Oct 11;24:660. doi: 10.1186/s13063-023-07685-3 (PMC10568883; doi:10.1186/s13063-023-07685-3)
Supplement: Supplementary file 1 — Additional file 1. The PREPARATION study investigators. [file 13063_2023_7685_MOESM1_ESM.docx]

**Additional file 1:** The PREPARATION study investigators

Koene van der Sloot, MD; Esther M. Dias, MD; Jasper E. Kal, MD, PhD; Marjolein C.O. van den Nieuwenhuyzen, MD, PhD; Manuela di Biase, MD; Martin Hagenaars, MD, PhD; Bies Oedairadjsingh, MD; Taco van den Ende, MD; Michel Timmerman, MD; Zjuul Segers, MD; Dominique H.P.A.M. Schoester, MD; Kristy M.J. Vons, MD; A. Filius, MD, PhD; Wim Van Harten, MD, PhD; Rudolf W. Poolman, MD, PhD; Michel M.P.J. Reijnen, MD, PhD; Peter G. Noordzij, MD, PhD: Barbara C. van Munster; MD, PhD
