# Supplementary material for: Is a preoperative multidisciplinary team meeting (cost)effective to improve outcome for high-risk adult patients undergoing noncardiac surgery: the PREPARATION study—a multicenter stepped-wedge cluster randomized trial
Source: Trials. 2023 Oct 11;24:660. doi: 10.1186/s13063-023-07685-3 (PMC10568883; doi:10.1186/s13063-023-07685-3)
Supplement: Supplementary file 2 — Additional file 2. Participating hospitals, all in The Netherlands. [file 13063_2023_7685_MOESM2_ESM.docx]

**Additional file 2**: **Participating hospitals, all in The Netherlands**

1. University Medical Center Groningen

2. Maastricht University Medical Center

3. Albert Schweitzer Hospital, Dordrecht

4. Bravis Hospital, Roosendaal

5. Canisius Wilhelmina Hospital, Nijmegen

6. Gelre Hospital, Apeldoorn

7. Groene Hart Hospital, Gouda

8. Ikazia Hospital, Rotterdam

9. Jeroen Bosch Hospital, ‘s-Hertogenbosch

10. Maxima Medical Center, Veldhoven

11. Meander Medical Center, Amersfoort

12. OLVG, Amsterdam

13. Zaans Medical Center, Zaandam

14. Zuyderland Medical Center, Heerlen
