# Supplementary material for: Is a preoperative multidisciplinary team meeting (cost)effective to improve outcome for high-risk adult patients undergoing noncardiac surgery: the PREPARATION study—a multicenter stepped-wedge cluster randomized trial
Source: Trials. 2023 Oct 11;24:660. doi: 10.1186/s13063-023-07685-3 (PMC10568883; doi:10.1186/s13063-023-07685-3)
Supplement: Supplementary file 3 — Additional file 3. WHO trial dataset. [file 13063_2023_7685_MOESM3_ESM.docx]

**Additional file 3: WHO trial dataset**

| Data category | Information |
| --- | --- |
| Primary registry and trial identifying number | Clinicaltrials.gov NCT05703230 |
| Date of registration in primary registry | 11 September 2022 |
| Secondary identifying numbers | Rijnstate: 2021-1983 ZonMW: 10330032010003 |
| Source(s) of monetary or material support | The Netherlands Organization for Health Research and Development (ZonMW) regarding the program ‘Healthcare Evaluation & Appropriate Use’. (file number: 10330032010003) |
| Primary sponsor | The Netherlands Organization for Health Research and Development (ZonMW) regarding the program ‘Healthcare Evaluation & Appropriate Use’. (file number: 10330032010003) |
| Secondary sponsor(s) | - |
| Contact for public queries | JV (jvernooij@rijnstate.nl) |
| Contact for scientific queries | NK CDo JV RB  Rijnstate Hospital, Arnhem, The Netherlands |
| Public title | *Is a preoperative multidisciplinary team meeting (cost)effective to improve outcome for high-risk adult patients undergoing noncardiac surgery:* ***The PREPARATION study.*** *A multicenter stepped-wedge cluster randomized trial* |
| Scientific title | *Is a preoperative multidisciplinary team meeting (cost)effective to improve outcome for high-risk adult patients undergoing noncardiac surgery:* ***The PREPARATION study.*** *A multicenter stepped-wedge cluster randomized trial* |
| Countries of recruitment | The Netherlands |
| Health condition(s) or problem(s) studied | Perioperative serious adverse events in high-risk noncardiac surgical patients. |
| Intervention(s) | Active comparator: *Preoperative multidisciplinary team discussion* |
|  | Placebo comparator: Care as usual |
| Key inclusion and exclusion criteria | Ages eligible for study: ≥18 years Sexes eligible for study: both Accepts healthy volunteers: no |
|  | • ≥18 years of age, and  • American Society of Anaesthesiology (ASA) physical status score ≥ 3, and  • Clinical Frailty Scale ≥ 4, and  • Planned for elective or semi-elective non-cardiac surgery, and  • As stated by the 2010 Dutch preoperative guideline:  o Doubt by the surgeon, anaesthesiologist or patient regarding the harm-benefit ratio of the surgical procedure; or  o Doubt if the correct measures were taken to limit the perioperative risk as much as possible; or  o Doubt if the patient agrees with the surgery and/or the anaesthesia plan |
|  | Exclusion criteria are:  • No informed consent  • Emergency surgery  • Impossible to communicate with the patient directly or through a third party, e.g. a relative or an interpreter  • Proposed surgical intervention for which a preoperative MDT meeting, similar to the current study intervention, already exists in that hospital at the start of the study |
| Study type | Interventional |
|  | Allocation: Stepped-wedge randomized cluster trial |
|  | Primary purpose: Reduction of inappropriate care |
|  |  |
| Date of first enrolment | November 2022 |
| Target sample size | 1200 |
| Recruitment status | Recruiting |
| Primary outcome(s) | The number of perioperative serious adverse events after six months |
| Key secondary outcomes | - Cost-effectiveness from a societal and healthcare perspective - Functional status of the patient at 3, 6 and 12 months - Patients’ experienced quality of life at 3,6 and 12 months - Patients’ regret about the decision at 3,6 and 12 months - The (self-assessed) performance and attendance of MDT meetings - Facilitators and barriers regarding the execution of MDT meetings |
