# Supplementary material for: Is a preoperative multidisciplinary team meeting (cost)effective to improve outcome for high-risk adult patients undergoing noncardiac surgery: the PREPARATION study—a multicenter stepped-wedge cluster randomized trial
Source: Trials. 2023 Oct 11;24:660. doi: 10.1186/s13063-023-07685-3 (PMC10568883; doi:10.1186/s13063-023-07685-3)
Supplement: Supplementary file 4 — Additional file 4. Tool box for implementation of multidisciplinary team meeting. [file 13063_2023_7685_MOESM4_ESM.docx]

**Additional file 4: Tool box for implementation of multidisciplinary team meeting**

A three-circle model (Figure 1) has been created to facilitate discussion during MDT meetings. This model combines treatment options, patient goals and preferences, and health situations to formulate a proper treatment plan during the meeting.

**Figure 1: Three circles model for personalized medicine**


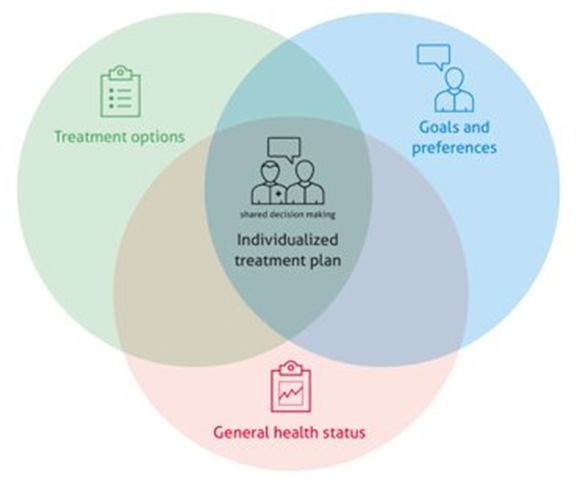


To be informed about the patient’s goals and preferences the Outcome Prioritization Tool (OPT) will be used. The OPT is a conversation aid with four visual analogue scales that each represents a universal health outcome: life extension, preserving independence, reducing pain and reducing other symptoms.

The toolbox also contains guiding questions that can be used during the MDT meeting to guide the multidisciplinary discussion. The questions include:

- What would be this patient’s approximate life expectancy, excluding/ignoring the indication for surgical intervention?
- How would the proposed surgical procedure or its alternative treatment options influence the patient’s life expectancy and burden of disease?
- What would be the treatment burden? (e.g., hospitalization duration, complication risk, outpatient ward visits)
- Is the proposed treatment in line with the patient’s goals and preferences?
- Is the harm/benefit ratio of the proposed surgery acceptable for this patient?
- Does the patient's health condition need to be optimized further before the proposed treatment can be instituted?
- What is the influence of the proposed alternative treatment and of alternatives to it on the patient's life expectancy and last illness?
