# Supplementary material for: Is a preoperative multidisciplinary team meeting (cost)effective to improve outcome for high-risk adult patients undergoing noncardiac surgery: the PREPARATION study—a multicenter stepped-wedge cluster randomized trial
Source: Trials. 2023 Oct 11;24:660. doi: 10.1186/s13063-023-07685-3 (PMC10568883; doi:10.1186/s13063-023-07685-3)
Supplement: Supplementary file 5 — Additional file 5. WHO trial registry data set. [file 13063_2023_7685_MOESM5_ESM.docx]

**Additional file 5:** **WHO trial registry data set**

| **Data category** |  |
| --- | --- |
| Primary registry and trial identifying number | ClinicalTrials.gov NCT05703230 |
| Date of registration in primary registry | September 11, 2022 |
| Secondary identifying numbers | Rijnstate: 2021-1983 ZonMW: 10330032010003 |
| Source(s) of monetary or material support | Grant received |
| Primary sponsor | The Netherlands Organization for Health Research and Development (ZonMW) regarding the program ‘Healthcare Evaluation & Appropriate Use’. (file number: 10330032010003) |
| Secondary sponsor(s) | - |
| Contact for public queries | PREPARATION@rijnstate.nl |
| Contact for scientific queries | jvernooij@rijnstate.nl |
| Public title | Effects of preoperative multidisciplinary meetings for high risk noncardiac surgical patients |
| Scientific title | Is a structured preoperative Multidisciplinary Team discussion (cost)effective in optimizing outcome for high-risk adult patients planned for noncardiac surgery: The PREPARATION study. A multi center stepped wedge randomized cluster design study. |
| Countries of recruitment | The Netherlands |
| Health condition(s) or problem(s) studied | High-risk noncardiac surgical patients |
| Intervention(s) | Preoperative Multidisciplinary Team discussion |
| Key inclusion and exclusion criteria | **Inclusion criteria:** patients ≥ 18 years of age, and with ASA physical status ≥3, and Critical Frailty Scale ≥4, and elective or semi-elective noncardiac surgery scheduled, and as stated by the Dutch perioperative guideline: Doubt regarding the harm-benefit ratio of the surgical procedure; or doubt if the correct measures were taken to limit the perioperative risk as much as possible; or doubt if the patient agrees with the surgery or the anesthesiologic treatment and the expected risks.  **Exclusion criteria**:   - Emergency surgery; - no informed consent; - no communication possible even with help of a relative; - “Proposed surgical intervention for which a structured preoperative multidisciplinary team meeting, similar to the current study intervention, already exists in this respective hospital at the start of the study” |
| Study type | Stepped wedge randomized cluster design study |
| Date of first enrolment | November first 2022 |
| Target sample size | 1120 patients |
| Recruitment status | Recruiting |
| Primary outcome(s) | Serious adverse events (SAEs) at 6 months, defined as:  1) Grade 3 or more on the Clavien Dindo classification following surgical intervention  2) non-operative SAEs will be graded accordingly i.e., events necessitating hospital admission, surgical intervention, single or  multi-organ failure, or death. |
| Key secondary outcomes | - Disability status assessed at timepoints as shown in table 2, measured by the 12-item WHO Disability Assessment Schedule  2.0 (WHODAS 2.0);  - Survival at 30 days 6 months, and 1 year postoperatively;  - Quality of life assessed by the Abbreviated World Health Organization Quality of Life WHOQOL BREF;  - Regret measured by a decision regret questionnaire;  - Facilitators and barriers to organize preoperative MDT meetings determined/evaluated using structured interviews with healthcare professionals from all different medical specialties;  - Societal costs over a time horizon of 12 months as measured by a cost questionnaire;  - The number and nature of alterations in perioperative management decided by the MDT;  - Multidisciplinary attendance of MDTs;  - Performance of MDTs using an MDT-Meeting Observational Tool (MDT-MODe);  - At baseline patients will be asked to prioritize their desired outcome with the Outcome Prioritization Tool (OPT);  - For every patient preoperative risk calculations will be completed using the surgical outcome risk tool (SORT), the revised  cardiac risk index for pre-operative risk (RCRI) and American college of surgeons ACS-NSQIP surgical risk calculator (SRC) |

**Protocol version**: 2.5; October 18, 2022

**Funding**: For this multicenter study financial support is granted by the largest public funding agency for health research in the Netherlands (ZONMW) file number: 10330032010003.

**Roles and responsibilities-contributor ship**: NK, CDi, CDo, BP, CK, BL, SF, HvdW, JvB and JV initiated the study design. JV and NK are grant holders. CDo and CK provided statistical expertise in clinical trial design and CDi is conducting the primary statistical analysis. All authors contributed to refinement of the study protocol and approved the final manuscript. JV conceived the first draft of the manuscript with help from RB.

**Roles and responsibilities-sponsor contact information**: File number: 10330032010003; The Netherlands Organization for Health Research and Development (ZonMW), application round Care Evaluation & Proper Use (ZE&GG) 2020.

Contact: Prof. Dr. Sjoerd Repping,

Care evaluation & appropriate care
Postbus 320
1110 AH Diemen

**Roles and responsibilities-sponsor and funder:** Prof. Dr. Sjoerd Repping gave advice on the study design at the start of the study. Prof. Dr. Sjoerd Repping will not have any further role during its execution, analyses, interpretation of the data, or decision to submit results.

**Roles and responsibilities-committees:**

**Principal investigator and project leader**:

Principal investigator: N.J. Koning, MD, PhD; project leader: J.E.M. Vernooij, MD, MA

- Design and conduct of PREPARATION
- Preparation of protocol and revisions
- Preparation of investigators brochure (IB) and CRFs [case report forms]
- Organising steering committee meetings
- Managing CTO [clinical trials office]
- Publication of study reports
- Members of TMC [Trial Management Committee]

**Steering committee (SC)**

Romijn Boerlage, MD; Carine J.M. Doggen, PhD; Benedikt Preckel, MD, PhD; Carmen D. Dirksen, PhD; Barbara L. van Leeuwen, MD, PhD; R.J. Spruit, MD; Suzanne Festen, MD, PhD; Hanneke van der Wal-Huisman, MSc; Jean P. van Basten, MD, PhD; Cor J. Kalkman, MD, PhD; Nick J. Koning, MD, PhD; Wim Van Harten, MD, PhD; Rudolf W. Poolman, MD, PhD; Michel M.P.J. Reijnen, MD, PhD; Peter G. Noordzij, MD, PhD: Barbara C. van Munster; MD, PhD; Jacqueline E.M. Vernooij, MD, MA.

- Agreement of final protocol
- Reviewing progress of study and if necessary agreeing changes to the protocol and/or investigators brochure to facilitate the smooth running of the study.

**Project group (PG)**

Romijn Boerlage, MD; Carine J.M. Doggen, PhD; Benedikt Preckel, MD, PhD; Carmen D. Dirksen, PhD; Barbara L. van Leeuwen, MD, PhD; Rutger J. Spruit, MD; Suzanne Festen, MD, PhD; Hanneke van der Wal-Huisman, MSc; Jean Paul van Basten, MD, PhD; Cor J. Kalkman, MD, PhD; Nick J. Koning, MD, PhD; Wim Van Harten, MD, PhD; Rudolf W. Poolman, MD, PhD; Michel M.P.J. Reijnen, MD, PhD; Peter G. Noordzij, MD, PhD: Barbara C. van Munster; MD, PhD; Jacqueline E.M. Vernooij, MD, MA.

**Collaborators:**

Koene van der Sloot, MD; Esther M. Dias, MD; Jasper E. Kal, MD, PhD; Marjolein C.O. van den Nieuwenhuyzen, MD, PhD; Manuela di Biase, MD, Martin Hagenaars, MD, PhD; Bies Oedairadjsingh, MD; Taco van den Ende, MD; Michel Timmerman, MD, PhD; Zjuul Segers, MD; Dominique H.P.A.M. Schoester, MD; Rutger J. Spruit, MD; Kristy M.J.Vons, MD; A. Filius, MD, PhD; All lead investigators will be project group members, as will all project committee members. One lead investigator per study hospital will be nominated as principal coordinator.

Recruitment of patients and liaising with principle investigator.

The Project Group will meet twice a year. (once virtually and once in real life)

**Trial management committee (TMC)**

(Principle investigator Nick J. Koning; project leader: Jacqueline E.M. Vernooij.

- Study planning
- Organisation of steering committee meetings
- Provide annual risk report and ethics committee
- SUSAR [Serious unexpected suspected adverse events] reporting to Ethics committee
- Responsible for trial master file and filing of protocol amendments if necessary
- Budget administration and contractual issues with individual centres
- Advice for lead investigators
- Assistance with international review, board/independent ethics committee applications
- Data verification
- Randomisation

**Data manager/PhDs/research nurse**

- Maintenance of trial IT system and data entry
- Data verification

**Lead investigators**

In each participating centre a lead investigator will be identified, to be responsible for identification, recruitment, data collection and completion of CRFs, along with follow up of study patients and adherence to study protocol and investigators brochure. Lead investigators will be projectgroup collaborators.
