# Supplementary material for: Is a preoperative multidisciplinary team meeting (cost)effective to improve outcome for high-risk adult patients undergoing noncardiac surgery: the PREPARATION study—a multicenter stepped-wedge cluster randomized trial
Source: Trials. 2023 Oct 11;24:660. doi: 10.1186/s13063-023-07685-3 (PMC10568883; doi:10.1186/s13063-023-07685-3)
Supplement: Supplementary file 7 — Additional file 7. [file 13063_2023_7685_MOESM7_ESM.docx]

**Deelnemer informatie (MDO) voor deelname aan onderzoek over groepsoverleg voor dat uw operatie plaatsvindt**

De PREPARATION studie

Geachte heer/mevrouw,

Met deze informatiebrief willen we u vragen of u wilt meedoen aan ons onderzoek. Meedoen is vrijwillig. Het onderzoek vindt plaats in verschillende ziekenhuizen in Nederland en wordt georganiseerd vanuit het Rijnstate ziekenhuis te Arnhem. In deze brief staat informatie over de PREPARATION studie: wat het voor u betekent, wat de voordelen en nadelen zijn.

Het is veel informatie. Na de uitleg door de onderzoeker, kunt u deze informatie rustig doorlezen. Daarnaast raden we u aan:

- Vragen te stellen aan degene die u deze informatie geeft,

- Te bespreken met uw partner, familie of vrienden of u zou willen deelnemen.

Als alles duidelijk is, kunt u besluiten of u wilt meedoen aan dit onderzoek. Als u wilt meedoen, kunt u het toestemmingsformulier invullen.

**Wat is het doel van het onderzoek?**

In het gesprek met uw behandelend arts over uw operatie heeft u te horen gekregen dat u kans heeft op het optreden van problemen tijdens of na de operatie. Wij onderzoeken het nut van een groepsoverleg tussen meerdere artsen en verpleegkundigen voor een operatie. Zij bespreken wat de beste behandeling is en welke voorzorgsmaatregelen er genomen kunnen worden. Dit doen ze zodat de kans op problemen zo laag mogelijk wordt. Daarbij wordt rekening gehouden met uw gezondheid en uw wensen. Het advies dat zij geven wordt door uw eigen arts met u besproken. Daarbij wordt rekening gehouden met uw eigen voorkeur voor een behandeling.

Wij willen onderzoeken welke problemen kunnen optreden. We willen dit vergelijken met een tijdsperiode waarin nog geen groepsoverleg plaats vond. Ook willen we nagaan hoe het tijdens het eerste jaar met u gaat. Daarnaast onderzoeken we de kosten voor de gezondheidszorg. Wij hopen door dit onderzoek in de toekomst de kans op problemen tijdens en na de operatie te verlagen. Ook hopen we de kwaliteit van leven te verbeteren.

**Wat betekent het voor u als u meedoet? Wat verwachten we van u?**

U krijgt gewoon alle zorg die u nodig heeft. We vragen u 4 keer een vragenlijst in te vullen. Deze vragenlijst gaat over uw gezondheid, uw voorkeur voor een behandeling, hoe u de behandeling ervaren heeft, en over hoe het met u gaat. U vult deze voor de operatie in, en 3, 6 en 12 maanden na de operatie. Het invullen kost ongeveer 30 minuten per keer. Als u de vragenlijst niet terugstuurt, wordt u gebeld. Indien nodig kunnen we u helpen met het invullen van de vragenlijst.

Voor het onderzoek zullen we ook gegevens over uw ziekte en behandeling uit uw medische gegevens in het ziekenhuis gebruiken. Mogelijk dat we ook gegevens bij uw huisarts of andere zorgverleners opvragen. We vragen hiervoor uw toestemming.

Voor het onderzoeken van de kosten van uw behandeling willen we uw gegevens over zorgkosten opvragen bij de zorgverzekeraar. Ook hiervoor vragen wij uw toestemming.

**Wat zijn de voordelen en nadelen als u meedoet?**

Doordat nu een groepsbespreking plaatsvindt wordt uw situatie uitgebreid besproken. Dit kan een voordeel zijn. Het kan zijn dat u na het gesprek met uw eigen arts voor een andere behandeling kiest. Het nadeel van meedoen aan dit onderzoek is dat u 4 keer een vragenlijst moet invullen.

**Wanneer stopt het onderzoek?**

Het onderzoek stopt voor u als:

- u de laatste vragenlijst na 12 maanden heeft ingevuld en teruggestuurd.
- u zelf wilt stoppen met het onderzoek. Dat mag op ieder moment. Meld dit dan meteen bij de onderzoeker. U hoeft er niet bij te vertellen waarom u stopt. U ontvangt vanaf dat moment geen vragenlijst meer.
- de onderzoeker het beter voor u vindt om te stoppen.

De onderzoekers gebruiken de gegevens die tot het moment van stoppen zijn verzameld.

Het totale onderzoek is afgelopen als alle deelnemers klaar zijn of als het onderzoek moet stoppen door een besluit van de overheid.

**Hoe gaan we om met uw gegevens?**

Doet u mee met het onderzoek? Dan geeft u toestemming om uw gegevens te verzamelen, gebruiken en bewaren.

We bewaren de volgende gegevens:

- uw naam

- uw geslacht

- uw adres

- uw email adres

- uw geboortedatum

- gegevens over uw gezondheid

- (medische) gegevens die we tijdens het onderzoek verzamelen

- gegevens uit de vragenlijsten.

**Waarom verzamelen, gebruiken en bewaren we uw gegevens?**

Uw gegevens worden gebruikt om de vragen van dit onderzoek te kunnen beantwoorden. U kunt uw toestemming voor het gebruik van uw gegevens op ieder moment weer intrekken.

**Hoe beschermen we uw privacy?**

Om uw privacy te beschermen krijgen uw gegevens een code. Uw gegevens zoals uw naam, adres of geboortedatum zijn bekend bij een kleine groep onderzoekers en projectleiders met wie u contact kunt hebben. Andere onderzoekers weten niet wie u bent. Ook in presentaties, rapporten en artikelen over het onderzoek zijn de gegevens niet naar u te herleiden. Alleen in uw eigen ziekenhuis en in Rijnstate ziekenhuis is de code aan uw naam gekoppeld.

**Wie kunnen uw gegevens wel zien?**

Sommige personen kunnen wel uw naam en andere persoonlijke gegevens inzien. Dit zijn mensen die controleren of de onderzoekers het onderzoek goed en betrouwbaar uitvoeren. De volgende personen kunnen bij uw gegevens komen:

- Een controleur die door Rijnstate is ingehuurd.
- Nationale en internationale toezichthoudende autoriteiten. Bijvoorbeeld de Inspectie Gezondheidszorg en Jeugd.
- De onderzoekers van Rijnstate en de onderzoekers uit uw eigen ziekenhuis.

Deze personen zijn verplicht uw gegevens geheim te houden. Zij hebben daarvoor getekend.

**Wat gebeurt er met uw gegevens na afloop van het onderzoek?**

Uw gegevens worden 15 jaar in uw ziekenhuis en in Rijnstate ziekenhuis bewaard. Uw gegevens kunnen na afloop van dit onderzoek mogelijk nog van belang zijn voor ander wetenschappelijk onderzoek naar operaties. In het toestemmingformulier kunt u aangeven of u dit opnieuw gebruiken goed vindt. Geeft u hiervoor geen toestemming? Dan kunt u nog steeds meedoen met dit onderzoek.

**Kunt u uw toestemming voor het gebruik van uw gegevens weer intrekken?**

Deelname aan het onderzoek is vrijwillig. U bent helemaal vrij om wel of niet mee te doen aan dit onderzoek. U kunt uw toestemming voor het gebruik van uw gegevens op ieder moment intrekken. Maar let op: trekt u uw toestemming in, en hebben onderzoekers dan al gegevens verzameld voor het onderzoek? Dan mogen zij deze gegevens nog wel gebruiken. Niet deelnemen aan het onderzoek of het intrekken van uw toestemming heeft geen enkel gevolg voor uw behandeling.

**Wilt u meer weten over uw privacy?**

- Wilt u meer weten over uw rechten bij de verwerking van persoonsgegevens? Kijk dan op <http://www.autoriteitpersoonsgegevens.nl>
- Heeft u vragen over uw rechten met betrekking tot de verwerking van uw gegevens? Neem dan contact op met de Functionaris voor Gegevensbescherming van uw eigen ziekenhuis [*naam eigen ziekenhuis invullen, telefoonnummer*] of van Rijnstate ziekenhuis via het telefoonnummer 06 11 29 96 57.
- Als u klachten heeft over de verwerking van uw persoonsgegevens, raden we u aan om deze eerst te bespreken met het onderzoeksteam. U kunt ook naar de Functionaris Gegevensbescherming van uw eigen ziekenhuis of van Rijnstate gaan. Of u dient een klacht in bij de Autoriteit Persoonsgegevens.

**Krijgt u een vergoeding voor dit onderzoek?**

U ontvangt geen vergoeding voor uw deelname. Het meedoen aan het onderzoek kost u zelf niets. U kunt de vragenlijst digitaal of via de post in een envelop zonder postzegel terugsturen.

**Bent u verzekerd?**

Er is voor dit wetenschappelijke onderzoek geen aparte verzekering afgesloten. Uiteraard is uw eigen ziektekostenverzekering normaal van kracht.

**Ontvangt u de resultaten van het onderzoek?**

Na afloop van het totale onderzoek, dat ongeveer 4 jaar zal duren, kunt u een samenvatting van de resultaten ontvangen. U kunt op het toestemmingsformulier aangeven of u dat wilt.

**Heeft u nog vragen?**

Bij vragen kunt u contact opnemen met de onderzoeker in uw ziekenhuis, op [*telnr invullen*]. U kunt ook terecht bij uw eigen arts. Uw eigen arts wordt ingelicht over uw deelname nadat u toestemming heeft gegeven. Wilt u advies van iemand die los staat van het onderzoek? U kunt dan terecht bij een onafhankelijke arts. Dat is dr. Arianne van Bon, bereikbaar via het telefoonnummer 088 00 56 73 5. Zij weet veel over het onderzoek, maar heeft er niets mee te maken. Heeft u een klacht? Bespreek dit dan met een van de onderzoekers of met uw eigen arts. Wilt u dit liever niet? Dan kunt u contact opnemen met de klachtenfunctionaris [*telefoonnr klachtenfunctionaris eigen ziekenhuis invullen*].

**Hoe geeft u toestemming?**

Denk rustig na over uw deelname. Deelname is vrijwillig. Wilt u meedoen? Vul dan het toestemmingsformulier in dat u bij deze informatiebrief vindt. U en de onderzoeker krijgen allebei een getekende versie van deze toestemmingsverklaring. Het formulier wordt ook bewaard in het archief van het onderzoek

Dank voor uw interesse in dit onderzoek.

Dr xxx [*Dr. Naam , functie, onderzoeker lokaal ziekenhuis invullen*]

Dr Nick Koning, Anesthesioloog, Hoofdonderzoeker Rijnstate Ziekenhuis Arnhem

Mede namens het gehele onderzoeksteam

**Bijlage bij deze informatie**

Bijlage A Toestemmingsformulier proefpersoon

**Bijlage A: Toestemmingsformulier proefpersoon**

**De PREPARATION studie**

- Ik ben akkoord met deelname aan dit onderzoek. Ik heb de informatiebrief gelezen en voldoende uitleg gekregen. Ik had genoeg tijd om te beslissen of ik meedoe.
- Ik weet dat meedoen vrijwillig is. Ook weet ik dat ik op ieder moment kan beslissen om te stoppen met deelname aan het onderzoek. Daarvoor hoef ik geen reden te geven.
- Ik geef toestemming aan de onderzoekers voor het verzamelen en gebruiken van mijn gegevens uit mijn medisch dossier. Deze gegevens worden uitsluitend gebruikt voor dit onderzoek.
- Ik geef toestemming aan de onderzoekers voor het opvragen van gegevens bij mijn huisarts of andere zorgverleners, indien nodig. Deze gegevens worden uitsluitend gebruikt voor dit onderzoek.
- Ik geef toestemming aan de onderzoekers voor het opvragen van mijn gegevens over zorgkosten bij zorgverzekeraars. Deze gegevens worden uitsluitend gebruikt voor dit onderzoek.
- Ik ga ermee akkoord dat de onderzoekers gecodeerde gegevens ontvangen. Zij weten niet wie ik ben. Alleen in mijn eigen ziekenhuis en in Rijnstate ziekenhuis Arnhem kunnen een klein aantal onderzoekers zien dat de gegevens van mij zijn. Deze onderzoekers hebben een geheimhoudingsplicht.
- Ik weet dat voor de controle van het wetenschappelijk onderzoek sommige mensen toegang tot mijn gegevens kunnen krijgen. Die mensen staan vermeld in de proefpersonen informatiebrief. Ik geef toestemming voor inzage door deze personen.
- Ik geef toestemming om mijn eigen behandelaar in te lichten over mijn deelname aan dit onderzoek.

**Kruis aan wat voor u van toepassing is**

Ik wil meedoen aan dit onderzoek.

□ ja

□ nee

Ik wil na afloop van het onderzoek graag een samenvatting ontvangen

□ ja

□ nee

Ik geef toestemming om mijn gegevens te bewaren en te gebruiken voor ander wetenschappelijk onderzoek naar operaties.

□ ja

□ nee

Naam proefpersoon: Geboortedatum:

_________________________ ____/_____/____

Emailadres _________________________

Thuisadres _________________________

Handtekening:

Datum: ____/_____/____

__________________________________________________________________________

Ik verklaar dat ik deze proefpersoon volledig heb geïnformeerd over het genoemde onderzoek.

Als er tijdens het onderzoek informatie bekend wordt die de toestemming van de proefpersoon zou kunnen beïnvloeden, dan breng ik hem/haar daarvan tijdig op de hoogte.

Naam onderzoeker (of diens vertegenwoordiger):

________________________

Handtekening:

Datum: ____/_____/____

Participant information (MDT) for participation in group consultation research before your operation is taking place: The PREPARATION study (translated by author)

Sir/Madam,

With this information letter we would like to ask you if you are willing to participate in our research. Participating is voluntary. The research will take place in various hospitals in the Netherlands and will be organized from the Rijnstate hospital in Arnhem. This letter contains information about the PREPARATION study: what it means for you, what the advantages and disadvantages are.

It's a lot of information. After the explanation by the researcher, you can take this information home and calmly read through. In addition, we recommend that you:

- Ask questions of the person who gives you this information,

- To discuss with your partner, family or friends whether you would like to participate.

If everything is clear, you can decide whether you want to participate in this study. If you want to participate, please complete the consent form.

What is the purpose of the research?

During the discussion with your treating physician about your operation, you were told that you have an higher risk of problems occurring during or after surgery. We investigate the usefulness of a group meeting between several doctors and nurses before an operation. They will discuss the best treatment options for you and what precautions can be taken to avoid complications. They do this so that the chance of problems is as low as possible. In doing so, they are taking into account your health and your wishes. The advice they give is passed on and discussed with you by your own doctor. Your own preference is taken into account in deciding on what treatment will be given. We want to investigate which problems can occur. We want to compare this to a period of time in which no group consultation took place. We also want to check how you are doing during the first year after the surgery. We also examine the costs for healthcare.

We hope that this research will reduce the risk of problems during and after surgery in the future for other patients as well. We also hope to improve the quality of life after surgery.

What does it mean to you if you participate? What do we expect from you?

You simply get all the care you need. We ask you to complete questionnaires 4 times.

This questionnaire is about your health, your treatment preferences, how much

treatment you got and how you are doing. You fill this in before the operation, and 3,

6 and 12 months after surgery. Filling in takes about 30 minutes each time. If you do not return the questionnaires, you will be called. If necessary, we can help you fill in the questionnaires.

For the research we will also collect information about your illness and treatment from your medical record using data in the hospital. We may also collect data from your General Practitioner or ask other health care providers. We ask for your permission for this.

We need your data to investigate the costs of your treatment and could request healthcare costs information from the health insurer. We also ask for your permission for this.

What are the advantages and disadvantages of participating?

Because a group discussion is going to take place, your situation will be discussed in detail. This is possibly an advantage. It may be that after talking to your own doctor you choose for another treatment. The disadvantage of participating in this study is that you have to complete questionnaires 4 times.

When does the investigation end?

The research will stop for you if:

• you have completed and returned the last questionnaire after 12 months.

• you want to stop the research yourself. That is allowed at any time. Then report this

directly to the researcher. You don't have to say why you're quitting. You receive no more questionnaires from that moment on.

• the researcher thinks it would be better for you to stop.

The researchers use the data collected up to the moment of stopping.

The total research is over when all participants have finished or when the research has to be terminated by government decision.

How do we handle your data?

Are you participating in the research? Then you give permission to collect, use and store your data.

We store the following data:

- your name

- your gender

- your address

- Your email address

- your date of birth

- information about your health

- (medical) data that we collect during the research

- data from the questionnaires.

Why do we collect, use and store your data?

Your data will be used to answer the questions of this research. You can withdraw your consent to the use of your data at any time.

How do we protect your privacy?

To protect your privacy, your data is assigned a code. Your data such as your name,

address or date of birth are known to a small group of researchers and project leaders with who you can contact. Other researchers do not know who you are. Also in presentations, reports and articles about the research, the data cannot be traced back to you. Only in your own hospital and in Rijnstate hospital the code is linked to your name.

Who can see your data?

Some people can see your name and other personal information. These are people who check whether the researchers conduct the research properly and reliably.

The following persons can access your data:

• An inspector hired by Rijnstate.

• National and international supervisory authorities. For example the Inspectorate for

Healthcare and Youth.

• The researchers from Rijnstate and the researchers from your own hospital.

These persons are obliged to keep your data confidential. They signed for it.

What happens to your data after the research has ended?

Your data will be stored in your hospital and Rijnstate hospital for 15 years. Your

data may still be of interest to others after the end of this study for scientific research on operations. In the consent form you can indicate whether you are happy to reuse it. Do you not give permission for this? Then you can still continue to participate in this research.

Can you withdraw your consent to the use of your data?

Participation in the research is voluntary. You are completely free to participate or not

to this research. You can give your consent to the use of your data and at any time

retract. But beware: if you withdraw your consent, researchers will then already have

data collected for the research. Then they can still use this data.

Not participating in the research or withdrawing your consent has no effect on your treatment.

Would you like to know more about your privacy?

• Would you like to know more about your rights when processing personal data? Look

then on http://www.autoriteitpersoonsgegevens.nl

• Do you have questions about your rights regarding the processing of your data?

Then please contact your own hospital Data Protection Officer:

[insert name of own hospital, telephone number] or van Rijnstate

hospital via telephone number 06 11 29 96 57.

• If you have any complaints about the processing of your personal data, we advise you

to discuss this with the research team first. You can also go to the Data Protection Officer of your own hospital or of Rijnstate. Or you can submit a complaint to the Dutch Data Protection Authority.

Will you be reimbursed for this research?

You will not receive any compensation for your participation. Participation in the research is at your own cost. You can return the questionnaire digitally or by mail in an envelope without a stamp.

Are you insured?

No separate insurance has been taken out for this scientific research. Of course

your own health insurance is normally in effect.

Do you receive the results of the investigation?

At the end of the total study, which will take about 4 years, you can receive a summary

of the results. You can indicate on the consent form whether you want this.

Do you have any questions?

If you have any questions, please contact the researcher at your hospital at [telephone number].

You can also consult your own doctor. Your own doctor will be informed about your participation after you have given permission. Would you like advice from someone separate from the investigation? You can contact an independent doctor. That is Dr. Arianne van Bon, who can be reached via the telephone number 088 00 56 73 5. She knows a lot about the research, but has nothing to do with it.

Do you have a complaint?

Then discuss this with one of the researchers or with your own doctor. Would you rather not? Then you can contact the complaints officer:

[enter telephone number of complaints officer at own hospital].

How do you give permission?

Please think carefully about your participation. Participation is voluntary. Do you want to participate? Then fill in the consent form enclosed with this information letter. You and the investigator get both a signed version of this consent form. The form is also kept in the archive of the research.

Thank you for your interest in this research!

Dr xxx [Dr. Fill in name, position, researcher local hospital]

Dr Nick Koning, Anesthesiologist, Principal Investigator Rijnstate Hospital Arnhem

Also on behalf of the entire PREPARATION research team.

Annex to this information

Appendix A Subject Consent Form for The PREPARATION study

- I agree to participate in this research. I have read the information letter and

received sufficient explanation. I had enough time to decide whether to participate.

- I know that participation is voluntary. I also know that I can decide at any time to

to stop participating in the study. I don't have to give a reason for that.

- I give permission to the researchers to collect and use my

data from my medical record. This data is used exclusively for this investigation.

- I give permission to the researchers to request data from my doctor or other health care providers, if necessary. These data are exclusive used for this research.

- I give permission to the researchers to request my data health insurance costs. This data is used exclusively for this research.

- I agree that the researchers receive encrypted data. They know not who I am. Only in my own hospital and in Rijnstate hospital Arnhem can a small number of researchers see that the data is mine. This researchers have a duty of confidentiality.

- I know that for the control of scientific research some people can access my data. Those people are listed in the subjects information letter. I give permission for inspection by these persons.

- I give permission to inform my own practitioner about my participation in this investigation.

Please tick what applies to you

I want to participate in this research.

□ yes

□ no

I would like to receive a summary at the end of the study

□ yes

□ no

I give permission to store and use my data for others scientific research on operations.

□ yes

□ no

Name of subject: Date of birth:

_________________________ ____/_____/____

E-mail address _________________________

Home address _________________________

Signature:

Date: ____/_____/____

I declare that I have fully informed this subject of the said research. If information becomes known during the investigation that requires the consent of the subject, I will inform him/her in good time.

Name of researcher (or his representative):

Signature:
